# Evaluation of the NucliSens EasyQ v2.0 Assay in Comparison with the Roche Amplicor v1.5 and the Roche CAP/CTM HIV-1 Test v2.0 in Quantification of C-Clade HIV-1 in Plasma

**DOI:** 10.1371/journal.pone.0103983

**Published:** 2014-08-26

**Authors:** Maximilian Muenchhoff, Savathee Madurai, Allison Jo Hempenstall, Emily Adland, Anna Carlqvist, Angeline Moonsamy, Manjeetha Jaggernath, Busisiwe Mlotshwa, Emma Siboto, Thumbi Ndung'u, Philip Jeremy Renshaw Goulder

**Affiliations:** 1 Department of Paediatrics, University of Oxford, Oxford, Oxfordshire, United Kingdom; 2 HIV Pathogenesis Programme, University of KwaZulu-Natal, Durban, KwaZulu-Natal, South Africa; 3 Global Clinical and Viral Laboratories, Amanzimtoti, KwaZulu-Natal, South Africa; 4 KwaZulu-Natal Research Institute for Tuberculosis and HIV (K-RITH), University of KwaZulu-Natal, Durban, South Africa; 5 Max Planck Institute for Infection Biology, Berlin, Berlin, Germany; 6 The Ragon Institute of Massachusetts General Hospital, Massachusetts Institute of Technology and Harvard University, Boston, Massachusetts, United States of America; University of the Witwatersrand, South Africa

## Abstract

Human immunodeficiency virus type 1 (HIV-1) genetic diversity poses a challenge to reliable viral load monitoring. Discrepancies between different testing platforms have been observed, especially for non-clade-B virus. Therefore we compare, in antiretroviral therapy (ART)-naïve South African subjects predominantly infected with HIV-1 clade-C, three commercially available assays: the COBAS AmpliPrep/COBAS TaqMan HIV-1 Test version 2.0 by Roche (CAP/CTM v2.0), the BioMérieux NucliSens Version 2.0 Easy Q/Easy Mag (NucliSens v2.0) and the Roche COBAS Amplicor HIV-1 Monitor Test Version 1.5 (Amplicor v1.5). Strong linear correlation was observed and Bland-Altman analyses showed overall good agreement between the assays with mean viral load differences of 0.078 log cp/ml (NucliSens v2.0 – Amplicor v1.5), 0.260 log cp/ml (CAP/CTM v2.0 – Amplicor v1.5) and 0.164 log cp/ml (CAP/CTM v2.0 – NucliSens v2.0), indicating lower mean viral load results for the Amplicor v1.5 and higher mean readings for the CAP/CTM v2.0. Consistent with observations following previous comparisons of CAP/CTM v2.0 versus Amplicor v1.5, the CAP/CTM v2.0 assay detected low-level viremia (median 65 cp/ml) in more than one-third of those in whom viremia had been undetectable (<20 cp/ml) in assays using the NucliSens platform. These levels of viremia are of uncertain clinical significance but may be of importance in early detection of ART resistance in those on treatment. Overall the three assays showed good comparability of results but with consistent, albeit relatively small, discrepancies for HIV-1 clade-C samples, especially in the low-viremic range that should be taken into account when interpreting viral load data.

## Introduction

In clinical care the plasma HIV RNA viral load is widely used as a marker of disease progression [1] and is crucial to monitor efficacy of antiretroviral therapy. Research studies in the field of HIV infection also rely upon accurate measurements of HIV RNA levels that are reproducible and comparable temporally and geographically across the broad range of genetic diversity of HIV-1. Since the emergence of the HIV epidemic several commercial nucleic acid amplification platforms have been developed to measure the viral load and are now available worldwide. As signal and nucleic acid amplification methods depend on sequence specific primers and probes, HIV subtype-specific polymorphisms in the target regions can affect hybridization and hence compromise the quantitative measurement. Recent studies have shown discrepancies between different assays, especially for non-clade B specimens [2]–[5].

Approximately 50% of all HIV-1-infections are caused by HIV-1 group M clade-C globally, whereas clade-A and clade-B HIV-infections as the second and third most prevalent subtypes account for only about 10% each [6]. This uneven distribution is driven by the fact that the vast majority of HIV infections are located in sub-Saharan Africa, where clade-C overwhelmingly predominates. More than 10 million people are living with HIV in this region. Most HIV research, and a disproportionate fraction of viral load measurements are undertaken outside of Africa, and have therefore focused mainly on non-C-clade samples. Thus, the C-clade strain is relatively underrepresented in most studies despite being the most relevant subgroup from a global perspective. In the published comparisons between the new generation viral load assays, the proportion of C-clade specimens is also underrepresented.

To address this gap, we performed a comparison between three different commercial testing platforms for HIV-1 viral load monitoring using predominantly clade-C samples from South Africa, the country with more HIV infections than any other. We conducted a direct comparison between the COBAS AmpliPrep/COBAS TaqMan HIV-1 Test version 2.0 by Roche (CAP/CTM v2.0), the BioMérieux NucliSens Version 2.0 Easy Q/Easy Mag (NucliSens v2.0) and the Roche COBAS Amplicor HIV-1 Monitor Test Version 1.5 (Amplicor v1.5) for HIV-1 RNA quantification in blood plasma from a cohort of HIV-1 clade-C-infected patients.

## Materials and Methods

### Study subjects and sample collection

In a cohort of 220 asymptomatic, HAART-naïve post-natal women attending a pediatric immunization clinic at the Prince Mshiyeni Memorial Hospital in Umlazi, Durban, South Africa between 2012–2013 (median age 26.3 yrs, median CD4 count 538/mm^3^, median VL 6,100 cp/ml), we had noted an unexpectedly high proportion with low viral loads using the NucliSens v2.0. We therefore selected a subset (n = 44), including samples selected to represent the range of viral loads observed, in order to determine whether, and if so to what degree, the NucliSens v2.0 was reading lower than the CAP/CTM v2.0. In a selected subset of 13 of these subjects, whose viral loads were low (<1000 cp/ml) using the NucliSens v2.0, samples were studied from two separate visits to the clinic in order to increase the power of the study for comparison in the low viremic range. Plasma was separated from EDTA whole blood and stored at −80°C until testing. Viral load measurements were performed on frozen samples without previous freeze-thaw cycles to ensure consistent sample viability for these two assays.

To compare these two currently available assays with the Amplicor v1.5 assay previously widely used, we selected a representative subset of samples (n = 38) that were collected between 2002–2005 from a similar cohort of antenatal women (n = 328, median age 27.3 yrs, median CD4 count 399/mm^3^, median viral load 29,350 cp/ml) recruited from Prince Mshiyeni Memorial Hospital and St Mary's Hospital in Durban, South Africa. In this cohort, viral loads had originally been determined in 2002–2005 from fresh EDTA plasma using the Amplicor v1.5 assay.

Overall, samples were studied from 82 different subjects. HIV-status was determined using the Determine and Uni-Gold HIV rapid tests. For those subjects with undetectable viral load results, HIV-status was confirmed by Western blot.

### Ethics statement

Written informed consent was obtained from all participating individuals, and the Biomedical Research Ethics Administration of the University of KwaZulu-Natal and the institutional review board at the University of Oxford approved the study.

### HIV-1 Subtype analysis

The HIV-1 genotype was determined by sequencing in 72 of the 82 subjects as described previously [7] and confirmed to be subtype C in all cases. The Gag sequence data used for this study were submitted to GenBank (GenBank accession numbers KJ948566-KJ948637).

### Viral load measurement

The three commercial kits were used to determine the viral loads of the samples according to the manufacturer's instructions at the Global Clinical Viral Laboratory, Durban, South Africa, a good clinical laboratory practice (GCLP) compliant laboratory accredited through the South African National Accreditation System (SANAS).

### COBAS AmpliPrep/COBAS TaqMan HIV-1 Test version 2.0 (CAP/CTM v2.0)

The CAP/CTM v2.0 uses the fully automated COBAS AmpliPrep Instrument for specimen processing and the COBAS TaqMan Analyzer for amplification and detection with a reported linear range of 20–10,000,000 cp/ml. The sample input was 850 µl EDTA-plasma for this study.

### NucliSens Version 2.0 Easy Q/Easy Mag (NucliSens v2.0)

The NucliSens v2.0 uses a p24 Gag targeted nucleic acid sequence-based amplification (NASBA) associated with molecular beacon probes for detection of amplified nucleic acid products. RNA was purified from 100 ul patient EDTA plasma using the fully automated EasyMag extractor. Subsequently isothermic cDNA amplification and detection was performed on the automated EasyQ instrument with a dynamic range of 100–10,000,000 cp/ml with a detection limit of 20 cp/ml.

### Roche COBAS Amplicor HIV-1 Monitor Test Version 1.5 (Amplicor v1.5)

The Amplicor v1.5 is an end-point RT-PCR assay targeting a consensus region in the *gag* gene. It was used as the standard (not the ultra-sensitive) assay with a linear range of 400–750,000 cp/ml using 200 ul of EDTA-plasma.

### Statistical Analysis

All results were converted to cp/ml and transformed to log cp/ml for further statistical analysis using GraphPad Prism v6.0. Samples with viral loads below the detection limit of the assay were assigned the value of the lower limit of detection, i.e. 20 cp/ml (log cp/ml = 1.30) for NucliSens v2.0 and CAP/CTM v2.0. To determine the linear relationship between the assays the Spearman correlation coefficient (r) was calculated. Bland-Altman analysis was used to assess the agreement between the different methods of viral load measurement [8]. The difference between the results for the same sample (a_1_–a_2_) was plotted against the average of the two measurements ((a_1_–a_2_)/2). The bias between the assays was calculated as the mean *m* of the difference between the two measurements (a_1_–a_2_) and its standard deviation (s) was calculated. The 95% limits of agreement between the assays were determined as m±1.96 s.

## Results

### Summary statistics

For some samples viral load measurements were only available for 2 out of the 3 platforms being compared, hence the number of analyzed samples varies between the different comparisons. Data were available for n = 35 samples for the comparison between Amplicor v1.5/NucliSens v2.0, n = 34 for Amplicor v1.5/CAP/CTM v2.0 and n = 88 for the CAP/CTM v2.0/NucliSens v2.0 comparison as shown in Table S1. The average viral loads and standard deviations as well as the results for the Bland-Altman analysis for the different comparisons are summarized in Table 1.

**Table 1:** Data summary for viral load results as determined by the Amplicor v1.5, NucliSens v2.0 and CAP/CTM v2.0 assay.

Comparison	n	Mean log VL (cp/ml)	SD of mean	Bland-Altman analysis		
		(95% CI)		Bias	SD of bias	95% Limits of Agreement
NucliSens v2.0	35	4.587 (4.301, 4.873)	0.8321	0.078	0.5349	−0.9705, 1.126
Amplicor v1.5	35	4.509 (4.201, 4.817)	0.8975			
CAP/CTM v2.0	34	4.702 (4.451, 4.953)	0.7188	0.2603	0.4783	−0.6771, 1.198
Amplicor v1.5	34	4.441 (4.120, 4.763)	0.9209			
CAP/CTM v2.0	88	3.584 (3.283, 3.886)	1.423	0.1642	0.4373	−0.6929, 1.021
NucliSens v2.0	88	3.420 (3.107, 3.733)	1.478			

### Performance of NucliSens v2.0 versus CAP/CTM v2.0

**Figure 1:**
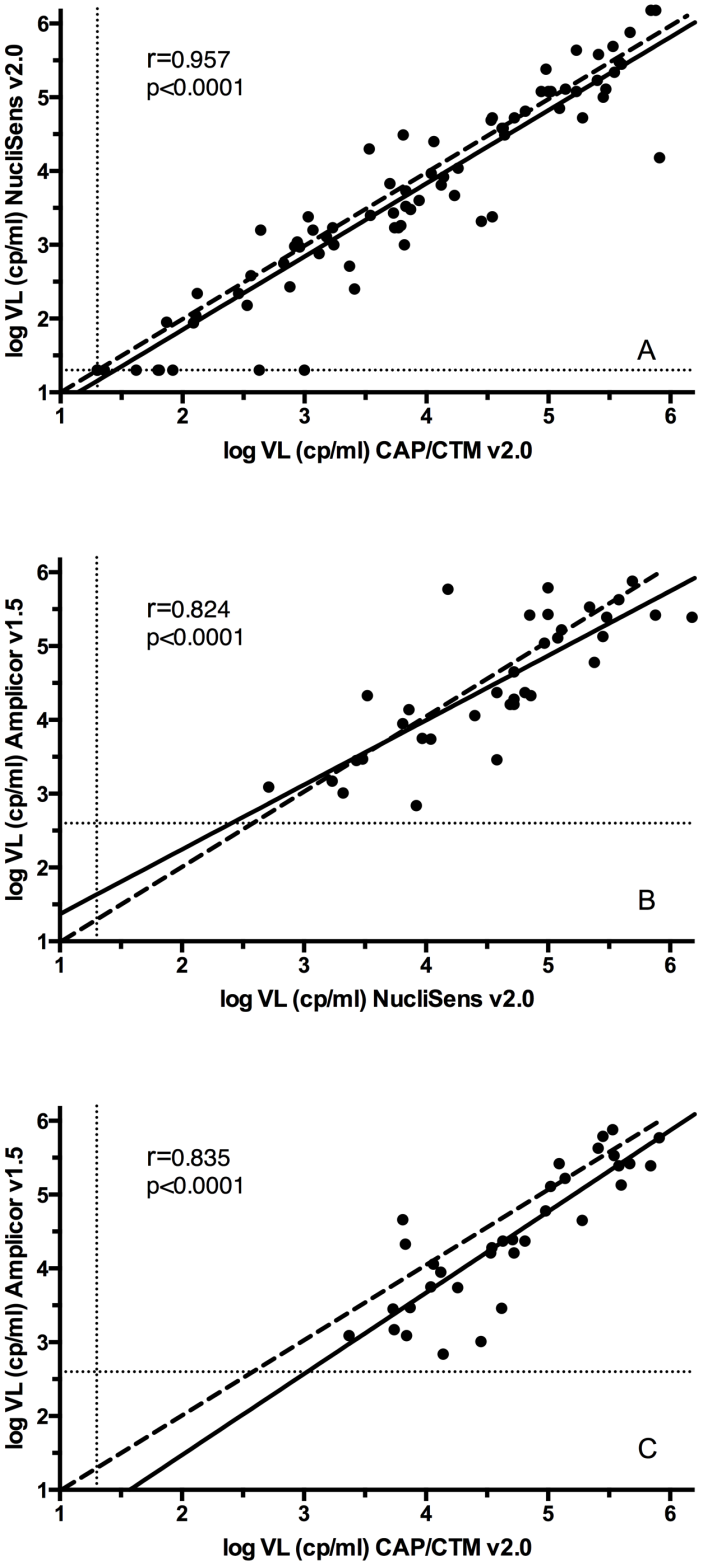
Linear correlation between (A) NucliSens v2.0 versus CAP/CTM v2.0 (B) NucliSens v2.0 versus Amplicor v1.5 (C) CAP/CTM v2.0 versus Amplicor v1.5. Solid lines represent the fitted linear regression curve, dashed lines show the equality line and dotted lines represent lower limits of detection of the respective assay.

The mean log viral load result for the n = 87 samples used for this comparison was 3.420 log cp/ml (3.107–3.733 log cp/ml) for the NucliSens v2.0 and 3.584 log cp/ml (3.283–3.886 cp/ml) for the CAP/CTM v2.0. In this dataset NucliSens v2.0 and CAP/CTM v2.0 showed a significant linear correlation (Spearman's correlation coefficient r = 0.957, p<0.0001, Figure 1A).

**Figure 2:**
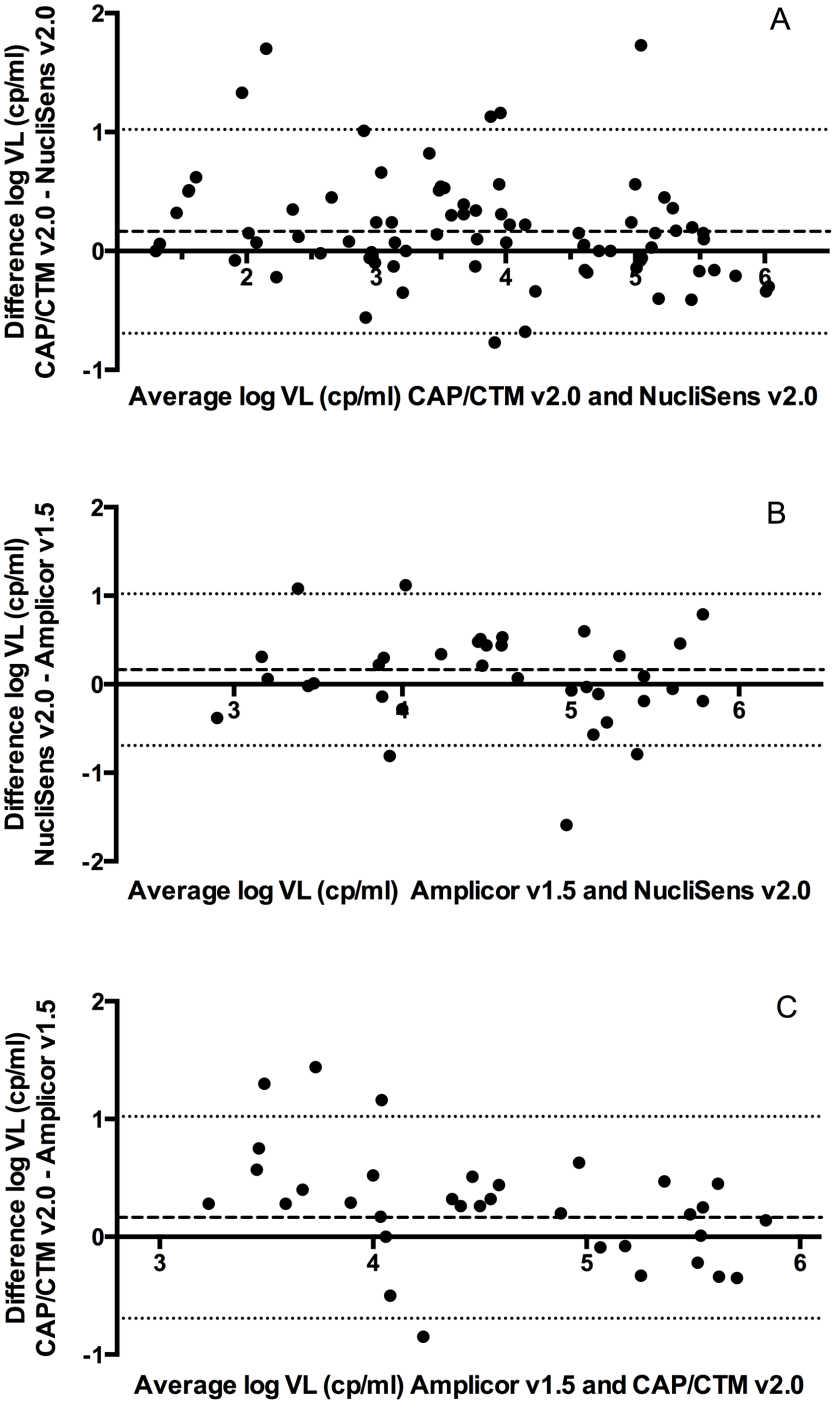
Bland-Altman model showing the agreement between (A) NucliSens v2.0 and CAP/CTM v2.0 (B) NucliSens v2.0 and Amplicor v1.5 (C) CAP/CTM v2.0 and Amplicor v1.5. Dashed horizontal lines show the mean difference and the dotted lines indicate the 95% limits of agreement.

In the Bland-Altman model (Figure 2A) the two assays showed high levels of agreement with a mean difference of m = 0.1642 log cp/ml s = 0.4373. Thus the NucliSens v2.0 tends to give slightly lower readings. Within this dataset there were 6 outliers (6.8% of samples) that were beyond the limits of agreement for this dataset, 5 of which read higher on the CAP/CTM v2.0. These outliers are evenly distributed throughout the range of viral loads. Of note, out of the 88 samples that were available for the comparison between the NucliSens v2.0 and CAP/CTM v2.0 assays, the output by the NucliSens v2.0 was <20 cp/ml (undetectable) for 18 samples. However, with the CAP/CTM v2.0 assay, a quantitative result was generated for 7 of these samples (Table S1), with a median viral load of 65 cp/ml as determined by the CAP/CTM v2.0. All samples that had a readout of <20 cp/ml on the CAP/CTM v2.0 also measured as <20 cp/ml on the NucliSens v2.0.

### Performance of NucliSens v2.0 versus Amplicor v1.5

For the n = 35 samples available for this comparison, the mean log viral load results were 4.587 log cp/ml (4.301–4.873 log cp/ml) for the NucliSens v2.0 and 4.509 cp/ml (4.201–4.817 log cp/ml) for the Amplicor v1.5. The assays showed significant linear correlation within the range of tested samples (r = 0.824, p<0.0001, Figure 1B). All analyzed samples were in the quantitative range of the assays.

Bland-Altman analysis showed close overall agreement between the assays with a bias of m = 0.078 log cp/ml (s = 0.5439) indicating slightly higher measurements for the NucliSens v2.0 compared to the Amplicor v1.5 (figure 2B). The 95% limits of agreement were −0.9705 and 1.126 log cp/ml showing 5 outliers (14.3% of samples) that did not follow a specific distribution throughout the range of viral loads that were represented in this comparison.

### Performance of CAP/CTM v2.0 versus Amplicor v1.5

Mean viral load results for the n = 34 samples analyzed for this comparison are 4.702 log cp/ml (4.451–4953 log cp/ml) for the CAP/CTM v2.0 and 4.441 log cp/ml (4.120–4.763 log cp/ml) for the Amplicor v1.5. Linear correlation across the analyzed log VL range that was within the quantitative limits of the two assays was strong (r = 0.835, p<0.0001).

In the Bland-Altman model the mean difference between the two assays was m = 0.2603 log cp/ml (s = 0.4783), reading higher on the CAP/CTM v2.0 with 95% limits of agreement of -0.6771 log cp/ml and 1.198 log cp/ml. Three of the 4 outliers (12% of samples) are in the lower log VL range and read higher on the CAP/CTM v2.0.

## Discussion

The reliable quantification of HIV-1 RNA viral load is a critical tool in HIV clinical care and research [9]. However, the high variability of the HIV-genome poses a great challenge to commercial assays to produce accurate measurements across the wide spectrum of HIV-1 subtypes. Previous studies have shown problems of under-estimation of viral load results by different testing platforms especially for non-B-clade samples [10]–[14].

Earlier studies evaluating the previous versions of the NucliSens assays, namely the NucliSens QT and its successors the NucliSens EasyQ v1.1 and v1.2, have shown underestimation of viral loads in non-clade-B samples [2], [5], [10], [14] and specifically clade-C samples [12], [13]. However, another study from South Africa found close agreement between the NucliSens EasyQ v1.1 and the Roche Amplicor v1.5 [4].

More recent studies evaluated the new version of the BioMerieux assay, the NucliSens v2.0. A study in China found improved performance in measuring non-B-clade samples [15], but no C-clade samples were included in this study. Another study tested different HIV-subtypes including 17 clade-C samples and found close agreement between the NucliSens v2.0 and the Abbott m2000 RealTime HIV-1 assay [11]. In a study from Gabon, the NucliSens v2.0 showed sub-optimal sensitivity in detecting circulating recombinant forms (CRFs) [16].

The need to adequately detect diverse HIV-subtypes has been appreciated and manufacturers are aiming to improve subtype inclusivity of their assays constantly. The latest version of the Roche platform, the CAP/CTM v2.0, therefore uses two dual-labeled hybridization probes that target both the *gag* and LTR regions to improve coverage of HIV-1 sequence polymorphisms [17]. Indeed, the sensitivity of this assay to detect non-B-clade virus has improved compared to its predecessor version and other methods including the NucliSens v1.2 and v2.0 and the Amplicor v1.5 [3], [5]. However, in the same studies the possibility of “over-quantification” due to the usage of two dual-labeled probes has been raised and is supported by showing constantly higher mean quantitative values for this assay compared to the other established methods. Yet, while the one study analyzed no HIV-1 clade-C samples at all, the other included only 1 clade-C sample.

We therefore performed this study in a South African cohort of HIV-1 clade-C infected subjects to compare recent versions of three widely used testing platforms, the CAP/CTM v2.0, NucliSens v2.0 and the Amplicor v1.5.

Our direct comparison between the NucliSens v2.0 and the Amplicor v1.5 has shown overall strong linear correlation (r = 0.824, p<0.0001) and agreement (mean difference, m = 0.078 cp/ml) with slightly higher readings on the NucliSens platform especially for samples in the range of 3–5 log cp/ml. The CAP/CTM v2.0 measured consistently higher than the Amplicor v1.5 with a bias of m = 0.2603 log cp/ml which is consistent with other studies [18], [19].

We also observed higher VL measurements for the CAP/CTM v2.0 compared with the NucliSens v2.0 with a mean difference of m = 0.1642 log cp/ml with strong linear correlation (r = 0.957, p<0.0001). This bias for clade-C virus between these two assays is lower than the difference that was observed in a study testing clade-B′, BC and AE samples from China measuring a mean difference of 0.588 log IU/ml [3]. Of note, for 7 of 18 samples defined as <20 cp/ml by the NucliSens v2.0 in our study, the CAP/CTM v2.0 generated measurements in the low-viremic range (median 65 cp/ml). Similar findings were observed in plasma from 109/502 C-clade infected subjects whose viral load was undetectable on the v1.5 assay, and detectable when retested using the v2.0 [18]. Other studies have also observed high sensitivity for the CAP/CTM v2.0 in the region of the lower limits of detection for different subtypes [20], [21].

Further factors to consider include the volume of sample required for the respective assays and costs. For this study we have used the minimum required sample input volume of 100 µl plasma for the NucliSens assay whereas 850 µl were used for the CAP/CTM v2.0. Lower sample volume requirements may be of particular interest in relation to pediatric studies. However, using the minimum volume for the NucliSens assay might compromise the sensitivity of the assay in the low-viremic range as stated by the manufacturer. At current prices the cost of the NucliSens assay is substantially lower than that of the CAP/CTM v2.0, a factor which is always of considerable relevance, but especially so in resource limited settings. It remains open to discussion whether the failure to detect low level viremia in a minority of those samples measuring <20 cp/ml on the NucliSens is of clinical significance and justifies increased costs of using the CAP/CTM v2.0 assay. Indeed, the introduction of novel more sensitive assays has led to an increase in low-positive viral load results of patients on antiretroviral therapy that has raised the question of which clinically relevant threshold should be used as an end-point for treatment efficacy [21]–[23].

It is important to note the limitations of this study. First, for the comparisons between the Amplicor v1.5 versus the CAP/CTM v2.0 and the NucliSens v2.0, the numbers studied in this dataset were restricted by sample availability, and thus those analyses were underpowered to draw strong conclusions. Second, none of the samples included in the comparisons with the Amplicor v1.5 had a viral load below 3 log cp/ml, and therefore this did not allow the performance of the Amplicor v1.5 to be assessed in the low viremic range. Third, plasma samples for the comparison with the Amplicor v1.5 were collected between 2002–2005 and run on the Amplicor v1.5 immediately but were stored at −80°C until run on the NucliSens v2.0 and CAP/CTM v2.0. The higher measurements on both of the two recent platforms compared to the Amplicor v1.5 might therefore be an underestimate. Finally, the low sample input volume of 100 µl for the NucliSens v2.0 assay may reduce the sensitivity of that assay, as mentioned above.

In conclusion, despite overall good agreement, there are discrepancies between the different testing platforms compared in this study using predominantly HIV-1 clade-C samples, especially in the low-viremic range. These differences have to be taken into account in clinical care, but also when comparing viral load results in clinical trials and other scientific studies. The decision to use one assay or the other, will certainly depend on the performance of the assay. But overall costs and throughput rates should also be considered, especially in resource limited settings.

## Supporting Information

Table S1 (DOCX)
