# Supplementary material for: Evaluation of the NucliSens EasyQ v2.0 Assay in Comparison with the Roche Amplicor v1.5 and the Roche CAP/CTM HIV-1 Test v2.0 in Quantification of C-Clade HIV-1 in Plasma
Source: PLoS One. 2014 Aug 26;9(8):e103983. doi: 10.1371/journal.pone.0103983 (PMC4144839; doi:10.1371/journal.pone.0103983)
Supplement: Table S1 — (DOCX) [file pone.0103983.s001.docx]

|  |  | **Viral load (log cp/ml)** | | |  |  |  | **Viral load (log cp/ml)** | | |
| --- | --- | --- | --- | --- | --- | --- | --- | --- | --- | --- |
| **Sample** | **Subject** | **Amplicor**  **v1.5** | **CAP/CTM**  **v2.0** | **Nuclisense**  **v2.0** |  | **Sample** | **Subject** | **Amplicor**  **v1.5** | **CAP/CTM**  **v2.0** | **Nuclisense**  **v2.0** |
| 1 | 1 | 2.84 | 4.14 | 3.92 |  | 49 | 44 | ND | <1.30 | <1.30 |
| 2 | 2 | 3.01 | 4.45 | 3.32 |  | 50 | 44 | ND | 1.36 | <1.30 |
| 3 | 3 | 3.09 | 3.37 | 2.71 |  | 51 | 45 | ND | 1.62 | <1.30 |
| 4 | 4 | 3.09 | 3.84 | ND |  | 52 | 45 | ND | 1.80 | <1.30 |
| 5 | 5 | 3.17 | 3.74 | 3.23 |  | 53 | 46 | ND | 1.81 | <1.30 |
| 6 | 6 | 3.45 | 3.73 | 3.43 |  | 54 | 46 | ND | 1.92 | <1.30 |
| 7 | 7 | 3.46 | 4.62 | 4.58 |  | 55 | 47 | ND | 2.63 | <1.30 |
| 8 | 8 | 3.47 | 3.87 | 3.48 |  | 56 | 47 | ND | 3.00 | <1.30 |
| 9 | 9 | 3.74 | 4.26 | 4.04 |  | 57 | 48 | ND | 2.09 | 1.94 |
| 10 | 10 | 3.75 | 4.04 | 3.97 |  | 58 | 48 | ND | 1.87 | 1.95 |
| 11 | 11 | 3.95 | 4.12 | 3.81 |  | 59 | 49 | ND | 2.11 | 2.04 |
| 12 | 12 | 4.06 | 4.06 | 4.40 |  | 60 | 49 | ND | 2.53 | 2.18 |
| 13 | 13 | 4.14 | ND | 3.86 |  | 61 | 50 | ND | 2.12 | 2.34 |
| 14 | 14 | 4.21 | 4.53 | 4.69 |  | 62 | 50 | ND | 2.46 | 2.34 |
| 15 | 15 | 4.21 | 4.72 | 4.72 |  | 63 | 51 | ND | 3.41 | 2.40 |
| 16 | 16 | 4.28 | 4.54 | 4.72 |  | 64 | 51 | ND | 2.88 | 2.43 |
| 17 | 17 | 4.33 | 3.83 | 3.52 |  | 65 | 52 | ND | 2.56 | 2.58 |
| 18 | 18 | 4.33 | ND | 4.86 |  | 66 | 53 | ND | 2.83 | 2.75 |
| 19 | 19 | 4.37 | 4.63 | 4.58 |  | 67 | 54 | ND | 3.12 | 2.88 |
| 20 | 20 | 4.37 | 4.81 | 4.81 |  | 68 | 55 | ND | 2.96 | 2.97 |
| 21 | 21 | 4.39 | 4.71 | ND |  | 69 | 56 | ND | 2.92 | 2.98 |
| 22 | 22 | 4.65 | 5.28 | 4.72 |  | 70 | 57 | ND | 3.82 | 3.00 |
| 23 | 23 | 4.66 | 3.81 | ND |  | 71 | 58 | ND | 3.24 | 3.00 |
| 24 | 24 | 4.78 | 4.98 | 5.38 |  | 72 | 59 | ND | 2.94 | 3.04 |
| 25 | 25 | 5.04 | ND | 4.97 |  | 73 | 60 | ND | 3.18 | 3.11 |
| 26 | 26 | 5.11 | 5.02 | 5.08 |  | 74 | 61 | ND | 2.64 | 3.20 |
| 27 | 27 | 5.13 | 5.60 | 5.45 |  | 75 | 62 | ND | 3.07 | 3.20 |
| 28 | 28 | 5.22 | 5.14 | 5.11 |  | 76 | 63 | ND | 3.23 | 3.23 |
| 29 | 29 | 5.39 | 5.58 | 5.48 |  | 77 | 64 | ND | 3.77 | 3.23 |
| 30 | 30 | 5.39 | 5.84 | 6.18 |  | 78 | 65 | ND | 3.79 | 3.26 |
| 31 | 31 | 5.42 | 5.09 | 4.85 |  | 79 | 66 | ND | 3.03 | 3.38 |
| 32 | 32 | 5.42 | 5.67 | 5.88 |  | 80 | 67 | ND | 4.54 | 3.38 |
| 33 | 33 | 5.43 | ND | 5.00 |  | 81 | 68 | ND | 3.54 | 3.40 |
| 34 | 34 | 5.53 | 5.54 | 5.34 |  | 82 | 69 | ND | 3.94 | 3.60 |
| 35 | 35 | 5.63 | 5.41 | 5.58 |  | 83 | 70 | ND | 4.23 | 3.67 |
| 36 | 36 | 5.77 | 5.91 | 4.18 |  | 84 | 71 | ND | 3.83 | 3.73 |
| 37 | 37 | 5.79 | 5.45 | 5.00 |  | 85 | 72 | ND | 3.70 | 3.83 |
| 38 | 38 | 5.88 | 5.53 | 5.69 |  | 86 | 73 | ND | 3.53 | 4.30 |
| 39 | 39 | ND | <1.30 | <1.30 |  | 87 | 74 | ND | 3.81 | 4.49 |
| 40 | 39 | ND | <1.30 | <1.30 |  | 88 | 75 | ND | 4.64 | 4.49 |
| 41 | 40 | ND | <1.30 | <1.30 |  | 89 | 76 | ND | 4.94 | 5.08 |
| 42 | 40 | ND | <1.30 | <1.30 |  | 90 | 77 | ND | 5.00 | 5.08 |
| 43 | 41 | ND | <1.30 | <1.30 |  | 91 | 78 | ND | 5.23 | 5.08 |
| 44 | 41 | ND | <1.30 | <1.30 |  | 92 | 79 | ND | 5.47 | 5.11 |
| 45 | 42 | ND | <1.30 | <1.30 |  | 93 | 80 | ND | 5.40 | 5.23 |
| 46 | 42 | ND | <1.30 | <1.30 |  | 94 | 81 | ND | 5.23 | 5.64 |
| 47 | 43 | ND | <1.30 | <1.30 |  | 95 | 82 | ND | 5.88 | 6.18 |
| 48 | 43 | ND | <1.30 | <1.30 |  |  |  |  |  |  |
